# Moderating Role of Anemia on the Association between Blood Urea Nitrogen and Atherosclerotic Cardiovascular Disease in Hypertension

**DOI:** 10.31083/RCM26245

**Published:** 2025-03-13

**Authors:** Qianqian Yu, Haitao Yu

**Affiliations:** ^1^Department of Blood Transfusion, The Second Affiliated Hospital of Anhui Medical University, 230601 Hefei, Anhui, China; ^2^Department of Critical Care Medicine, The First Affiliated Hospital of Anhui Medical University, 230022 Hefei, Anhui, China

**Keywords:** hypertension, anemia, blood urea nitrogen, atherosclerotic cardiovascular disease, moderating effect

## Abstract

**Background:**

Anemia or blood urea nitrogen (BUN) are both associated with atherosclerotic cardiovascular disease (ASCVD) in hypertension (HTN). However, the relationship between anemia, BUN, and ASCVD remains unclear in HTN. This study aimed to investigate the relationship between BUN, anemia, and ASCVD in HTN patients, and further investigated the moderating effect of anemia on the relationship between BUN and ASCVD.

**Methods:**

In total, 15,109 HTN patients were included based on the National Health and Nutritional Examination Survey (NHANES) from 1999 to 2018. The weighted univariate logistic regression model was utilized to select potential covariates. The relationship between BUN, anemia, and ASCVD was investigated using weighted univariate and multivariate logistic regression models. All results were expressed as odds ratios (ORs) and 95% confidence intervals (CIs).

**Results:**

A total of 15,109 HTN patients were included for final analysis. BUN level ≥4.69 mmol/L was related to higher odds of ASCVD in HTN patients (OR = 1.68, 95% CI: 1.51–1.88). Similarly, anemia was also associated with increased odds of ASCVD in HTN patients (OR = 1.45, 95% CI: 1.22–1.73). In patients with anemia, a BUN level ≥4.69 mmol/L was associated with increased odds of ASCVD when compared to patients who had a BUN level <4.69 mmol/L (OR = 2.95, 95% CI: 2.05–4.25). Anemia affected the association between BUN and ASCVD in HTN patients.

**Conclusions:**

Anemia moderates the association between BUN and ASCVD in HTN patients, amplifying the adverse effects. The findings show the importance of comprehensive management strategies that included renal function monitoring and anemia treatment in HTN patients.

## 1. Introduction

Hypertension (HTN) is a highly prevalent chronic disease globally, with its prevalence increasing with advancing age. HTN is a significant risk factor for atherosclerotic cardiovascular disease (ASCVD), potentially leading to higher mortality rates [1]. However, despite the use of antihypertensive therapy to control blood pressure, there remains a residual cardiovascular risk among HTN patients [2]. Therefore, it is crucial to accurately identify other potential risk factors and implement appropriate management strategies to mitigate the risk of ASCVD.

The kidney plays a vital role in regulating blood pressure and the onset of HTN [3]. Blood urea nitrogen (BUN), a protein metabolic waste product produced by the liver and excreted by the kidneys, is used as a biomarker for periodic assessment of renal function. BUN is also associated with neurohormonal activation, and an elevation in BUN reflects the cumulative effect of hemodynamic and neurohormonal changes, leading to inadequate renal perfusion, oxidative stress, and an increased risk of atherosclerosis [4, 5]. In addition, BUN can reflect the relationship between nutritional status, protein metabolism, and renal function, making it an important marker for metabolic diseases and the nutritional status of patients [6]. High BUN levels independently predict all-cause mortality in heart failure [7]. Nevertheless, the association between BUN and ASCVD risk in HTN has not been explored.

Anemia is a significant global health issue, diagnosed based on the World Health Organization (WHO) criteria when hemoglobin (Hb) levels fall below 13 g/dL in males and 12 g/dL in females [8]. In hypertensive patients, antihypertensive medications can reduce hemoglobin levels and result in anemia through mechanisms such as blood thinning and suppression of erythropoiesis [9]. A cohort study previously showed that anemia increases the risk of cardiovascular and renal events in hypertensive patients with well-controlled blood pressure [10]. Additionally, previous research identified an interaction between anemia and impaired renal function, contributing to poor prognosis in heart failure patients. Therefore, our study aimed to investigate the association between BUN, anemia, and the risk of ASCVD in middle-aged hypertensive patients, while also exploring the moderating role of anemia on the association between BUN and ASCVD risk.

## 2. Methods

### 2.1 Study Design and Participants

The National Health and Nutritional Examination Survey (NHANES) is a nationally representative survey of non-institutionalized USA civilian populations conducted by the National Center for Health Statistics (NCHS) using a complex, multistage probability sampling design. All participants completed a household survey, which included questions on demographics and health history, as well as a physical examinations and blood sample testing. Details of study implementation are available for online access to NHANES Questionnaires, Datasets, and Related Documentation (https://wwwn.cdc.gov/nchs/nhanes/). Informed consent was obtained from all participants before the data collection, and the survey was approved by the NCHS Research Ethics Review Board. The data used in NHANES were de-identified to maintain confidentiality, ensuring compliance with ethical standards for research involving human subjects. The requirement of ethical approval for this study was waived by the Institutional Review Board of the First Affiliated Hospital of Anhui Medical University because the data was accessed from NHANES (a publicly available database).

In this cross-sectional study, data on HTN patients were extracted from the NHANES database from 1999 to 2018. Initially, 22,250 hypertensive patients aged 40 to 79 were enrolled. Subsequently, 4359 patients with a history of cardiovascular disease (CVD) were excluded. Additional exclusions were made for patients missing data on BUN measurement, hemoglobin levels, energy intake, body mass index (BMI), and ASCVD. Hypertension was defined by any of the following criteria: self-reported HTN previously diagnosed by healthcare professionals, use of antihypertensive medications, or elevated biological measurements (systolic blood pressure ≥130 mmHg and/or diastolic blood pressure ≥80 mmHg) [2].

### 2.2 Blood Urea Nitrogen

BUN was measured according to the fasting non-hemolytic samples from the subjects. The LX20 modular chemistry (BUNm) was used to quantitatively determine the concentration of blood urea nitrogen in serum or plasma using the enzymatic conductivity rate method. In our study, BUN was classified into two groups based on the median.

### 2.3 Anemia Assessment

Anemia was defined using the WHO criteria, in which a hemoglobin level <13 g/dL in males and <12 g/dL in females was defined as anemia [8].

### 2.4 ASCVD Assessment

Ten-year ASCVD risk scores can be calculated according to the ASCVD Risk Estimator (https://tools.acc.org/ldl/ascvd_risk_estimator/index.html#!/calulate/estimator/). The score combined sex- and race-specific algorithms to predict 10-year absolute ASCVD risk. Risk estimates were based on age, blood pressure, total cholesterol, high-density lipoprotein cholesterol, diabetes, smoking, and treatment of HTN. Individuals aged 40 to 79 with no previous diagnosis of CVD were eligible for ASCVD risk score calculation.

### 2.5 Covariates

Sociodemographic variables included age, race (white, black, other), gender (male and female), education level (less than 9th grade, 9–11th grade, high school grade, some college or Associate of Arts (AA) degree, college graduate or above), marriage status (married, widowed, divorced, separated, never married, living with partner, unknown) and poverty-to-income ratio (PIR). Behavioral characteristics included smoking, alcohol consumption, and physical activity. Health factors included BMI, diabetes, hyperlipidemia, and chronic kidney disease (CKD). Dietary information recorded total energy intake, dietary iron intake, and dietary quality index which was measured by the dietary approaches to stop hypertension. Laboratory measures included white blood cell (WBC) count, lymphocytes, neutrophils, platelets, uric acid, albumin, urea nitrogen, and hemoglobin levels.

### 2.6 Statistical Analysis

All analyses weighted the sample data with weights from the sdmvpsu, sdmvstra, and vtmec2yr variables in the NHANES database. Descriptive statistics are used to analyze the characteristics of the population, with quantitative data described as mean and standard error (S.E.) and qualitative data described as numbers and percentages (%). The differences between the high and low ASCVD risk groups were measured by applying the weighted *t*-test and chi-square test, respectively. The potential covariates were selected by using the weighted univariate logistic regression models. Model 1 was a crude model. Model 2 adjusted for BMI, education, marital status, PIR, drink status, physical activity, CKD, WBC, neutrophil count, platelet, uric acid, and energy. Weighted univariate and multivariable logistic regression models were used to explore the relationship between anemia, BUN, and ASCVD in hypertensive patients. The moderating effect of anemia on the association between BUN and ASCVD was further investigated. The results were presented as odds ratios (ORs) and 95% confidence intervals (CIs). All analyses were conducted using SAS 9.4 (SAS Institute Inc., Cary, NC, USA) and *p* < 0.05 was considered statistically different.

## 3. Results

### 3.1 Basic Characteristic of Participants

**Fig. 1.**
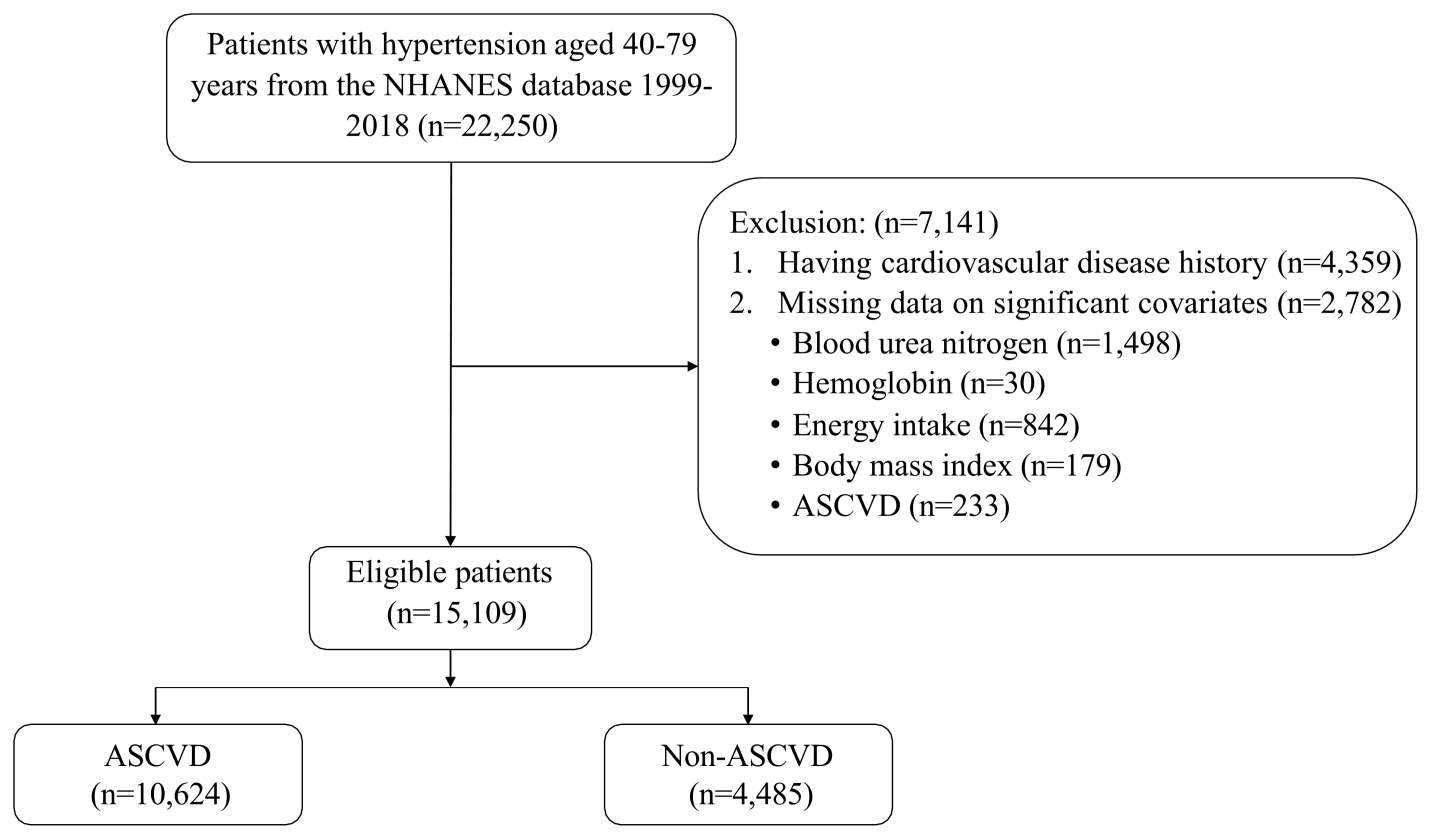
**Flow chart of the included HTN patients**. NHANES, National Health and Nutritional Examination Survey; HTN, hypertension; ASCVD, atherosclerotic cardiovascular disease.

Fig. 1 shows the selection process of included HTN patients. Initially, 22,250 HTN patients aged 40–79 years were included. Then, 4359 patients were excluded as they had a history of CVD. Then, patients were excluded from those missing data on BUN measurement (n = 1498), hemoglobin (n = 30), energy intake (n = 842), BMI (n = 179), and ASCVD (n = 233). In total, 15,109 HTN patients were included for final analysis. The basic characteristics of HTN patients are shown in Table 1. Statistical differences were found between the ASCVD and non-ASCVD groups in age, race, gender, education, marital status, PIR, smoking status, drinking status, physical activity, BMI, diabetes, dyslipidemia, CKD, cancer, protein, white blood cell count, neutrophils number, platelet count, uric acid, albumin, energy, blood urea nitrogen, and anemia.

**Table 1.** **Characteristics of HTN patients**.

Variables		Total (n = 15,109)	ASCVD		Statistics	*p*
			<20% (n = 10,624)	≥20% (n = 4485)		
Age (year), mean (S.E.)		56.85 (0.13)	53.71 (0.12)	68.48 (0.17)	*t* = –78.03	<0.001^#^
Race, n (%)					χ^2^ = 30.849	<0.001^*^
	White	6480 (72.71)	4545 (72.75)	1935 (72.55)		
	Black	3624 (11.31)	2396 (10.69)	1228 (13.59)		
	Other	5005 (15.98)	3683 (16.56)	1322 (13.86)		
Gender, n (%)					χ^2^ = 214.22	<0.001^*^
	Male	7477 (48.92)	4617 (45.44)	2860 (61.84)		
	Female	7632 (51.08)	6007 (54.56)	1625 (38.16)		
Education, n (%)					χ^2^ = 178.753	<0.001^*^
	Less than 9th grade	2094 (5.94)	1226 (4.76)	868 (10.30)		
	9–11th grade	2125 (10.41)	1412 (9.58)	713 (13.49)		
	High school grade	3625 (25.62)	2531 (25.07)	1094 (27.66)		
	Some college or AA degree	4099 (30.42)	3047 (31.39)	1052 (26.80)		
	College graduate or above	3166 (27.61)	2408 (29.19)	758 (21.74)		
Marital Status, n (%)					χ^2^ = 293.098	<0.001^*^
	Married	9006 (64.83)	6322 (65.27)	2684 (63.19)		
	Widowed	1395 (6.86)	676 (4.72)	719 (14.80)		
	Divorced	2145 (13.42)	1590 (14.02)	555 (11.21)		
	Separated	564 (2.53)	447 (2.79)	117 (1.57)		
	Never married	1215 (7.16)	974 (7.78)	241 (4.85)		
	Living with partner	638 (4.23)	515 (4.49)	123 (3.26)		
	Unknown	146 (0.98)	100 (0.94)	46 (1.13)		
PIR, n (%)					χ^2^ = 32.336	<0.001^*^
	<1.3	3673 (14.86)	2480 (14.09)	1193 (17.72)		
	≥1.3	10,115 (78.14)	7245 (79.29)	2870 (73.86)		
	Unknown	1321 (7.00)	899 (6.62)	422 (8.42)		
Smoking status, n (%)					χ^2^ = 483.864	<0.001^*^
	No	7850 (51.68)	6339 (57.18)	1511 (31.27)		
	Yes	7259 (48.32)	4285 (42.82)	2974 (68.73)		
Drinking status, n (%)					χ^2^ = 18.806	<0.001^*^
	No	4095 (22.64)	2910 (22.06)	1185 (24.77)		
	Often drink	3971 (31.20)	2891 (32.19)	1080 (27.52)		
	Sometimes drink	5158 (33.40)	3482 (32.98)	1676 (34.98)		
	Unknown	1885 (12.76)	1341 (12.77)	544 (12.74)		
Physical activity (MET·min/week), n (%)					χ^2^ = 37.220	<0.001^*^
	<450	6479 (42.45)	4505 (42.26)	1974 (43.15)		
	≥450	6277 (43.98)	4623 (45.04)	1654 (40.05)		
	Unknown	2353 (13.57)	1496 (12.70)	857 (16.80)		
BMI, n (%)					χ^2^ = 9.704	0.002^*^
	<25	3086 (20.71)	2216 (21.28)	870 (18.58)		
	≥25	12,023 (79.29)	8408 (78.72)	3615 (81.42)		
Diabetes, n (%)					χ^2^ = 914.035	<0.001^*^
	No	11,410 (81.02)	9011 (87.51)	2399 (56.98)		
	Yes	3699 (18.98)	1613 (12.49)	2086 (43.02)		
Dyslipidemia, n (%)					χ^2^ = 83.420	<0.001^*^
	No	2693 (17.41)	2150 (19.20)	543 (10.78)		
	Yes	12,416 (82.59)	8474 (80.80)	3942 (89.22)		
CKD, n (%)					χ^2^ = 281.248	<0.001^*^
	No	12,901 (88.62)	9478 (91.42)	3423 (78.26)		
	Yes	2208 (11.38)	1146 (8.58)	1062 (21.74)		
Cancer, n (%)					χ^2^ = 125.263	<0.001^*^
	No	13,595 (88.53)	9806 (90.88)	3789 (79.82)		
	Yes	1514 (11.47)	818 (9.12)	696 (20.18)		
Protein, g, n (%)					χ^2^ = 30.896	<0.001^*^
	<RDA	3561 (19.83)	2321 (18.65)	1240 (24.18)		
	≥RDA	11,548 (80.17)	8303 (81.35)	3245 (75.82)		
White blood cell count, mean (S.E.)		7.24 (0.03)	7.19 (0.04)	7.41 (0.05)	*t* = –3.65	<0.001^#^
Lymphocyte number, mean (S.E.)		2.12 (0.01)	2.12 (0.01)	2.11 (0.03)	*t* = 0.46	0.650^#^
Neutrophils number, mean (S.E.)		4.30 (0.02)	4.26 (0.03)	4.43 (0.03)	*t* = –4.37	<0.001^#^
Platelet count, mean (S.E.)		253.60 (0.87)	257.01 (0.97)	240.98 (1.55)	*t* = 9.32	<0.001^#^
Uric acid (mg/dL), mean (S.E.)		5.61 (0.02)	5.53 (0.02)	5.93 (0.03)	*t* = –10.41	<0.001^#^
Albumin (g/L), mean (S.E.)		42.51 (0.05)	42.61 (0.06)	42.16 (0.08)	*t* = 5.85	<0.001^#^
Energy (kcal), mean (S.E.)		2121.98 (11.05)	2167.56 (13.28)	1953.00 (16.44)	*t* = 9.98	<0.001^#^
Iron (mg), mean (S.E.)		17.13 (0.17)	17.11 (0.20)	17.18 (0.29)	*t* = –0.18	0.855^#^
DASH, mean (S.E.)		2.50 (0.02)	2.48 (0.02)	2.54 (0.04)	*t* = –1.36	0.175^#^
Blood urea nitrogen (mmol/L), mean (S.E.)		5.14 (0.02)	4.96 (0.02)	5.81 (0.05)	*t* = –17.60	<0.001^#^
Blood urea nitrogen (mmol/L), n (%)					χ^2^ = 151.414	<0.001^*^
	<4.74	7361 (47.48)	5693 (50.82)	1668 (35.09)		
	≥4.74	7748 (52.52)	4931 (49.18)	2817 (64.91)		
Hemoglobin (g/dL), mean (S.E.)		14.38 (0.03)	14.37 (0.03)	14.41 (0.04)	*t* = –1.21	0.227^#^
Anemia, n (%)					χ^2^ = 33.435	<0.001^*^
	No	13,766 (94.06)	9795 (94.68)	3971 (91.78)		
	Yes	1343 (5.94)	829 (5.32)	514 (8.22)		

^#^*t* test; ^*^ chi-square test. S.E., standard error; HTN, hypertension; ASCVD, atherosclerotic cardiovascular disease; PIR, poverty-to-income ratio; BMI, body mass index; CKD, chronic kidney disease; RDA, recommended dietary allowance; DASH, dietary approaches to stop hypertension; MET, metabolic equivalent of task.

### 3.2 Association between BUN, Anemia and ASCVD in HTN Patients

Table 2 presents the associations between BUN, anemia, and ASCVD in HTN patients. After adjusting for BMI, education, marital status, PIR, drinking status, physical activity, CKD, cancer, white blood cell count, neutrophil number, platelet count, uric acid, total energy intake, and protein, BUN level ≥4.69 mmol/L was related to a higher odds of ASCVD in HTN patients (OR = 1.68, 95% CI: 1.51–1.88). Similarly, anemia was also associated with increased odds of ASCVD in HTN patients (OR = 1.45, 95% CI: 1.22–1.73). The individuals with higher BUN and anemia (OR = 1.13, 95% CI: 1.03–1.24) were related to increased odds of ASCVD in patients with HTN.

**Table 2.** **Associations between BUN, anemia and ASCVD in HTN patients**.

Variables		Model 1		Model 2	
		OR (95% CI)	*p*	OR (95% CI)	*p*
BUN					
	<4.69	Ref		Ref	
	≥4.69	1.91 (1.73–2.12)	<0.001	1.68 (1.51–1.88)	<0.001
Anemia					
	No	Ref		Ref	
	Yes	1.59 (1.36–1.87)	<0.001	1.45 (1.22–1.73)	<0.001
BUN*Anemia		1.26 (1.15–1.38)	<0.001	1.13 (1.03–1.24)	<0.001

OR, odds ratio; CI, confidence interval; Ref, references; HTN, hypertension; ASCVD, atherosclerotic cardiovascular disease; BUN, blood urea nitrogen; *, the multiplicative interaction effect of BUN and anemia. Model 1: Crude model. Model 2: Adjusting body mass index, education, marital status, poverty income ratio, drinking status, physical activity, chronic kidney disease, cancer, white blood cell count, neutrophil count, platelet, uric acid, energy, and protein.

### 3.3 Anemia Affecting the Relationship between BUN and ASCVD

**Fig. 2.**
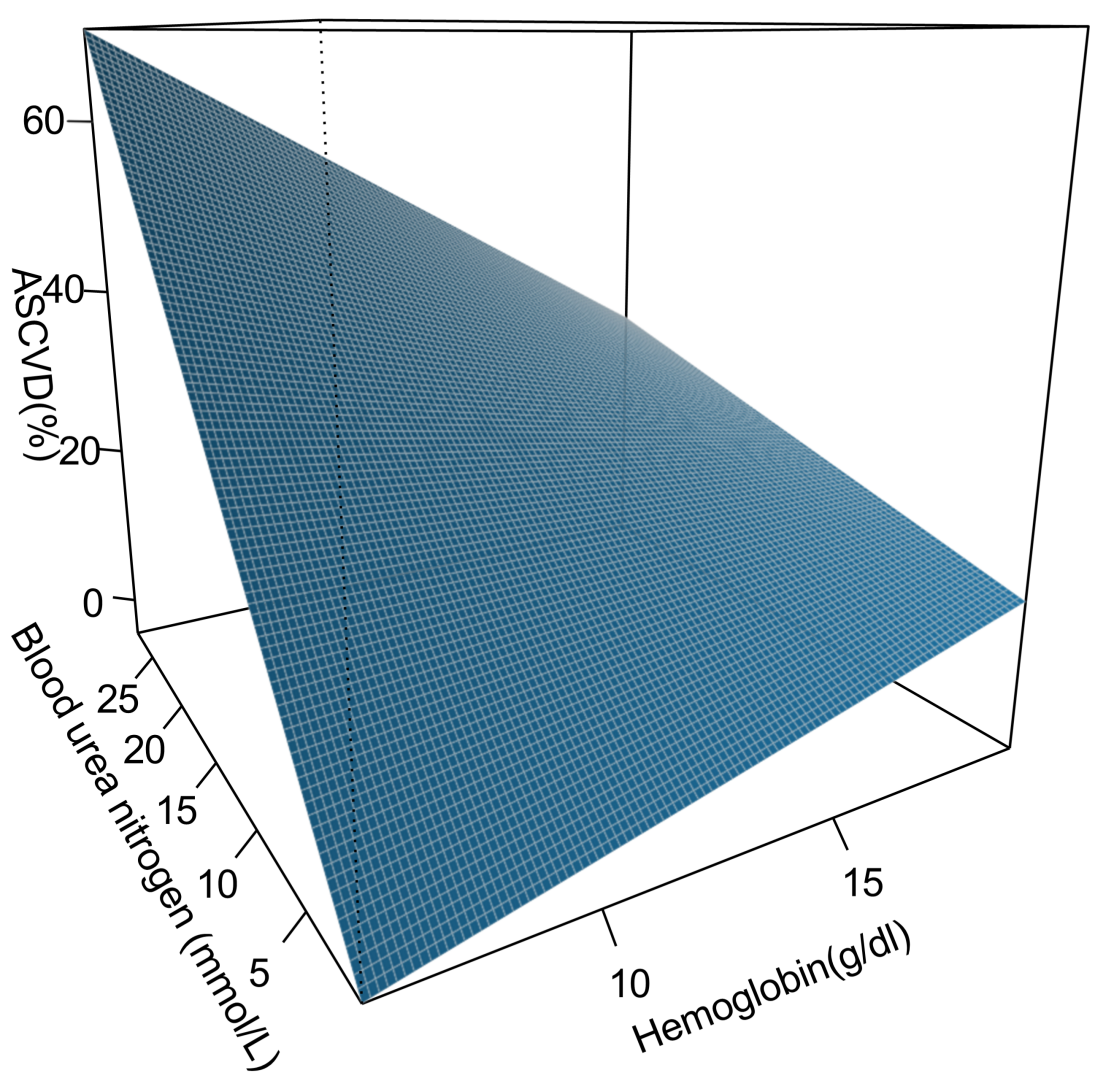
**Moderating effect of anemia on the relationship between BUN and ASCVD in HTN patients**. ASCVD, atherosclerotic cardiovascular disease; HTN, hypertension; BUN, blood urea nitrogen.

We further investigated the moderating effect of anemia on the relationship between BUN and ASCVD in HTN patients (Table 3). In the adjusted model 2, BUN level ≥4.69 mmol/L was associated with higher odds of ASCVD in HTN patients without anemia (OR = 1.62, 95% CI: 1.45–1.83). In patients with anemia, a BUN level ≥4.69 mmol/L was associated with increased odds of ASCVD when compared to patients with a BUN level <4.69 mmol/L (OR = 2.95, 95% CI: 2.05–4.25). Fig. 2 depicts that with an increasing Hb level, the ASCVD risk in HTN patients with elevated BUN levels is gradually decreasing. The findings indicated that anemia affected the association between BUN and ASCVD in HTN patients.

**Table 3.** **Moderating role of anemia on the relationship of BUN with ASCVD in HTN patients**.

Variables		Model 1		Model 2	
		OR (95% CI)	*p*	OR (95% CI)	*p*
Anemia	BUN				
No	<4.69	Ref		Ref	
	≥4.69	1.80 (1.61–2.00)	<0.001	1.62 (1.45–1.83)	<0.001
Yes	<4.69	Ref		Ref	
	≥4.69	4.51 (3.22–6.31)	<0.001	2.95 (2.05–4.25)	<0.001

OR, odds ratio; CI, confidence interval; Ref, references; ASCVD, atherosclerotic cardiovascular disease; HTN, hypertension; BUN, blood urea nitrogen. Model 1: Crude model. Model 2: Adjusting body mass index, education, marital status, poverty income ratio, drinking status, physical activity, chronic kidney disease, cancer, white blood cell count, neutrophil count, platelet, uric acid, energy, and protein.

### 3.4 Association between BUN, Anemia and ASCVD in Different Subgroups

We further investigated the associations of BUN and anemia with ASCVD in patients with HTN. The modifying effect of anemia on the association between BUN and ASCVD was also found in those ages <65 (OR = 2.28, 95% CI: 1.34–3.89), males (OR = 2.64, 95% CI: 1.54–4.53), females (OR = 2.65, 95% CI: 1.54–4.57), without CKD (OR = 3.21, 95% CI: 2.21–4.67), with CKD (OR = 2.59, 95% CI: 1.30–5.15), without cancer (OR = 2.78, 95% CI: 1.97–3.94), with cancer (OR = 5.63, 95% CI: 1.71–18.60), and protein intake less than (OR = 2.01, 95% CI: 1.12–3.59) and no less than (OR = 3.89, 95% CI: 2.51–6.01) recommended dietary allowance (Table 4).

**Table 4.** **Association between BUN, anemia, and ASCVD in different subgroups**.

Variables		Non-anemia		Anemia		Non-Anemia		Anemia	
		OR (95% CI)	*p*	OR (95% CI)	*p*	OR (95% CI)	*p*	OR (95% CI)	*p*
		Age <65				Age ≥65			
Blood urea nitrogen									
	<4.69	Ref		Ref		Ref		Ref	
	≥4.69	1.01 (0.79–1.28)	0.961	2.28 (1.34–3.89)	0.003	1.12 (0.92–1.35)	0.260	1.44 (0.79–2.62)	0.230
		Males				Females			
Blood urea nitrogen									
	<4.69	Ref		Ref		Ref		Ref	
	≥4.69	1.47 (1.24–1.73)	<0.001	2.64 (1.54–4.53)	<0.001	1.69 (1.42–2.02)	<0.001	2.65 (1.54–4.57)	<0.001
		Non-CKD				CKD			
Blood urea nitrogen									
	<4.69	Ref		Ref		Ref		Ref	
	≥4.69	1.70 (1.48–1.95)	<0.001	3.21 (2.21–4.67)	<0.001	1.33 (1.01–1.76)	0.045	2.59 (1.30–5.15)	0.008
		Non-Cancer				Cancer			
Blood urea nitrogen									
	<4.69	Ref		Ref		Ref		Ref	
	≥4.69	1.65 (1.46–1.87)	<0.001	2.78 (1.97–3.94)	<0.001	1.54 (1.13–2.09)	0.006	5.63 (1.71–18.60)	0.006
		Protein < RDA				Protein ≥ RDA			
Blood urea nitrogen									
	<4.69	Ref		Ref		Ref		Ref	
	≥4.69	1.78 (1.42–2.23)	<0.001	2.01 (1.12–3.59)	0.019	1.58 (1.37–1.81)	<0.001	3.89 (2.51–6.01)	<0.001

Adjusting body mass index, education, marital status, poverty income ratio, drinking status, physical activity, chronic kidney disease, cancer, white blood cell count, neutrophil count, platelet, uric acid, energy, and protein. ASCVD, atherosclerotic cardiovascular disease; BUN, blood urea nitrogen; OR, odds ratio; CI, confidence interval; CKD, chronic kidney disease; RDA, recommended dietary allowance; Ref, references.

## 4. Discussion

Our study investigated the relationship between anemia, BUN, and ASCVD in HTN patients based on NHANES data from 1999 to 2018. The findings suggested that elevated BUN levels and anemia were associated with increased odds of ASCVD in HTN patients. Moreover, anemia could affect the relationship between BUN level and ASCVD in HTN patients.

Consistent with our findings, the association between BUN and ASCVD has been reported [4, 6]. Jujo *et al*. [11] reported that persistently elevated BUN was associated with an increased number of CVD events. Moreover, BUN can act as an independent predictor of mortality in patients [12, 13]. The underlying mechanism for this association is likely multifactorial. Firstly, elevated BUN levels may signify impaired renal function, which has been established as a risk factor for ASCVD [14, 15, 16]. Secondly, elevated BUN levels may serve as an indicator of increased sympathetic nervous system activity and activation of the renin-angiotensin-aldosterone system, both of which contribute to the pathogenesis of HTN and ASCVD [17, 18].

Anemia was also associated with an increased incidence of ASCVD. Gan *et al*. [19] demonstrated a bidirectional causal relationship between anemia and heart failure. Treating anemia in heart failure patients could improve symptoms and long-term outcomes [20]. Anemia can result in reduced oxygen-carrying capacity, leading to tissue hypoxia and increased cardiac workload. Chronic tissue hypoxia stimulates erythropoiesis and increases blood viscosity, further straining the cardiovascular system [21, 22]. Additionally, anemia is often associated with chronic inflammation, which promotes atherosclerosis through various mechanisms, including endothelial dysfunction, lipid accumulation, and plaque destabilization [23].

Anemia affects the association between BUN and ASCVD in HTN patients, suggesting that anemia exacerbates the adverse effects of elevated BUN levels on the incidence of ASCVD. Anemia and hypertension are risk factors for renal prognosis and survival in diabetic patients [24]. Some explanations can be given for the modulating effects of anemia. Firstly, anemia aggravated tissue hypoxia and reduced oxygen delivery to the kidneys, allowing uremic toxins to accumulate and accelerating the progression of CVD in patients. Meanwhile, anemia increases oxidative stress in patients with CKD, producing excessive reactive oxygen species and nitrogen to accelerate disease progression [25]. Secondly, HTN patients are often in a state of persistent low-grade inflammation, and anemia induces the production of inflammatory cytokines [interleukin (IL)-1, IL-6, and tumor necrosis factor-alpha], which lead to endothelial dysfunction and poor outcomes [26, 27]. Finally, anemia-related hemodynamic alterations. Anemia can trigger compensatory mechanisms, including increased cardiac output and peripheral vasoconstriction. These adaptions can increase the workload on the heart and promote the development of left ventricular hypertrophy and CVD outcomes such as heart failure [28].

Hypertensive patients with elevated BUN levels are at increased odds of ASCVD, which is further enhanced by the presence of anemia. Therefore, clinicians should consider BUN and Hb levels simultaneously when assessing the incidence of ASCVD in hypertensive patients. Regular assessment of renal function is crucial in identifying individuals at higher risk. Early detection and treatment of anemia should be incorporated into the comprehensive management strategy for hypertensive patients, aiming to improve oxygen delivery and alleviate the cardiovascular burden. However, further prospective studies are needed to establish causality and explore potential therapeutic interventions.

Our study has some strengths. First, this is a population-based study with a large sample size. Second, to our knowledge, it is the first to report the moderating effect of anemia on the relationship between BUN and ASCVD in hypertensive patients. However, some limitations should be acknowledged. The cross-sectional nature of the study limited the exploration of the causal relationship between anemia, BUN, and ASCVD in HTN patients. Randomized controlled trials or prospective cohort studies are necessary to identify causal relationships. Furthermore, considering HTN and ASCVD tend to occur in middle-aged and elderly people, we limited patients to those aged ≥40 years. The association still needs to be verified in young HTN patients.

## 5. Conclusions

Our study suggests that there is anassociation between elevated BUN levels, anemia, and ASCVD risk in hypertensive patients. Anemia moderates the association between BUN and ASCVD, amplifying the adverse effects. The mechanisms underlying our findings involved impaired oxygen delivery, chronic inflammation, and bidirectional interactions between HTN, BUN, and ASCVD. Our results emphasize the importance of comprehensive management strategies that include regular monitoring of renal function and early treatment of anemia in hypertensive patients with elevated BUN. Further research is needed to establish causality and explore potential therapeutic interventions in hypertension patients.

## Availability of Data and Materials

The datasets generated and/or analyzed during the current study are available in the NHANES database, https://wwwn.cdc.gov/nchs/nhanes/.
